# *Xanthomonas hortorum* – beyond gardens: Current taxonomy, genomics, and virulence repertoires

**DOI:** 10.1111/mpp.13185

**Published:** 2022-01-23

**Authors:** Nay C. Dia, Lucas Morinière, Bart Cottyn, Eduardo Bernal, Jonathan M. Jacobs, Ralf Koebnik, Ebrahim Osdaghi, Neha Potnis, Joël F. Pothier

**Affiliations:** ^1^ Environmental Genomics and Systems Biology Research Group Institute for Natural Resource Sciences Zurich University of Applied Sciences Wädenswil Switzerland; ^2^ Molecular Plant Breeding Institute of Agricultural Sciences ETH Zurich Zurich Switzerland; ^3^ University of Lyon Université Claude Bernard Lyon 1 CNRS INRAE UMR Ecologie Microbienne Villeurbanne France; ^4^ Plant Sciences Unit Flanders Research Institute for Agriculture, Fisheries and Food Merelbeke Belgium; ^5^ Department of Plant Pathology The Ohio State University Columbus Ohio USA; ^6^ Infectious Diseases Institute The Ohio State University Columbus Ohio USA; ^7^ Plant Health Institute of Montpellier University of Montpellier, CIRAD, INRAe, Institut Agro, IRD Montpellier France; ^8^ Department of Plant Protection College of Agriculture University of Tehran Karaj Iran; ^9^ Department of Entomology and Plant Pathology Auburn University Alabama USA

**Keywords:** bacterial blight, carrot, dandelion, leaf spots, lettuce, pelargonium, tomato, *Xanthomonas hortorum*

## Abstract

**Taxonomy:**

Bacteria; Phylum *Proteobacteria*; Class *Gammaproteobacteria*; Order *Lysobacterales* (earlier synonym of *Xanthomonadales*); Family *Lysobacteraceae* (earlier synonym of *Xanthomonadaceae*); Genus *Xanthomonas*; Species *X*. *hortorum*; Pathovars: pv. *carotae*, pv. *vitians*, pv. *hederae*, pv. *pelargonii*, pv. *taraxaci*, pv. *cynarae*, and pv. *gardneri*.

**Host range:**

*Xanthomonas hortorum* affects agricultural crops, and horticultural and wild plants. Tomato, carrot, artichoke, lettuce, pelargonium, ivy, and dandelion were originally described as the main natural hosts of the seven separate pathovars. Artificial inoculation experiments also revealed other hosts. The natural and experimental host ranges are expected to be broader than initially assumed. Additionally, several strains, yet to be assigned to a pathovar within *X*. *hortorum*, cause diseases on several other plant species such as peony, sweet wormwood, lavender, and oak‐leaf hydrangea.

**Epidemiology and control:**

*X. hortorum* pathovars are mainly disseminated by infected seeds (e.g., *X*. *hortorum* pvs *carotae* and *vitians*) or cuttings (e.g., *X*. *hortorum* pv. *pelargonii*) and can be further dispersed by wind and rain, or mechanically transferred during planting and cultivation. Global trade of plants, seeds, and other propagating material constitutes a major pathway for their introduction and spread into new geographical areas. The propagules of some pathovars (e.g., *X*. *horturum* pv. *pelargonii*) are spread by insect vectors, while those of others can survive in crop residues and soils, and overwinter until the following growing season (e.g., *X*. *hortorum* pvs *vitians* and *carotae*). Control measures against *X*. *hortorum* pathovars are varied and include exclusion strategies (i.e., by using certification programmes and quarantine regulations) to multiple agricultural practices such as the application of phytosanitary products. Copper‐based compounds against *X*. *hortorum* are used, but the emergence of copper‐tolerant strains represents a major threat for their effective management. With the current lack of efficient chemical or biological disease management strategies, host resistance appears promising, but is not without challenges. The intrastrain genetic variability within the same pathovar poses a challenge for breeding cultivars with durable resistance.

**Useful websites:**

https://gd.eppo.int/taxon/XANTGA, https://gd.eppo.int/taxon/XANTCR, https://gd.eppo.int/taxon/XANTPE, https://www.euroxanth.eu, http://www.xanthomonas.org, http://www.xanthomonas.org/dokuwiki

## Graphical abstract

This pathogen profile summarizes the knowledge on the seven pathovars of *Xanthomonas hortorum* that cause bacterial spot and/or blight on 65 plant species in 15 families, including agricultural, horticultural, and wild plants.

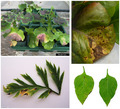

## 1 INTRODUCTION

The seven pathovars of *Xanthomonas hortorum* collectively affect 65 plant species in 15 botanical families, including agricultural crops (e.g., tomato, carrot, lettuce), horticultural plants (e.g., pelargonium), and wild plants (e.g., dandelion). This pathogen profile gives the first comprehensive summary of *X*. *hortorum* biology, including a history of its taxonomy and an account of its broad host range, and of its distribution and epidemiology, emphasizing intrapathovar differences. The genomics work done on this species is also summarized, with a special focus on pathogen–host interactions. Most previous literature on this pathogen deals with *X*. *hortorum* as a homogenous entity. This pathogen profile highlights, for each section, intrapathovar similarities and differences, thus providing a nuanced and detailed look into the complexity of *X*. *hortorum*.

## 2 TAXONOMY UPDATE

**FIGURE 1:**
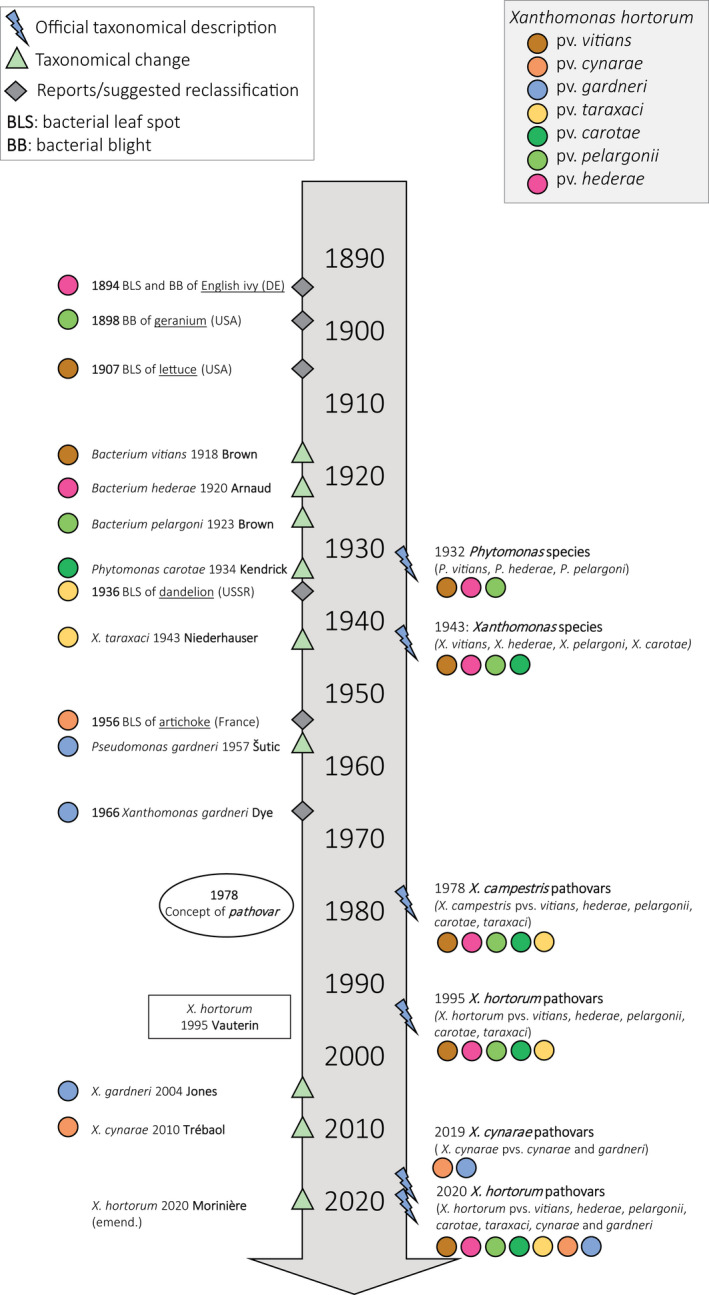
The taxonomical history of *Xanthomonas hortorum*, outlining official taxonomical descriptions and changes, as well as first reports or suggested reclassifications of the various pathovars

The taxonomic history of the different *X*. *hortorum* pathovars is long and complex (Figure 1), like that of the genus *Xanthomonas*. The earliest reports of diseases caused by *X*. *hortorum* date back to the 1890s, with the reports describing bacterial leaf spot and blight disease of English ivy in 1894 in Germany (Lindau, 1894), and bacterial blight of geraniums and bacterial leaf spot of lettuce in Massachusetts, USA, in 1898 and 1907, respectively (Stone, 1907; Stone & Smith, 1898). The first proper taxonomic description of the species causing bacterial leaf spot of lettuce, referred to as *Bacterium vitians* (Brown, 1918), was published in 1918 (Figure 1). In the following years, *B. hederae* and *B. pelargonii* were isolated from diseased English ivy (Arnaud, 1920) and diseased geraniums (Brown, 1923), respectively. *B. vitians*, *B*. *hederae*, and *B*. *pelargoni* were then reclassified in the genus *Phytomonas* (Bergey et al., 1923; Burkholder & Guterman, 1932). *Phytomonas carotae* (originally proposed as “*Pseudomonas carotae*”) was characterized as the bacterium responsible for bacterial blight of carrot (Kendrick, 1934).

Subsequently, the four species were transferred to the genus *Xanthomonas* as *X*. *hederae*, *X*. *carotae*, *X*. *pelargonii*, and *X*. *vitians* (Dowson, 1943; Starr & Burkholder, 1942). Concurrently, the bacterium responsible for the bacterial blight of Russian dandelion, first reported in the USSR (Sigrianski, 1936), was designated as *X. taraxaci* (Niederhauser, 1943). Those five pathogens were considered to be individual *Xanthomonas* species until the introduction of the infrasubspecific epithet “pathovar” (Young et al., 1978), followed by the publication of the first Approved Lists in 1980 (Skerman et al., 1980). Many *Xanthomonas* species, including *X*. *hederae*, *X*. *carotae*, *X*. *pelargonii*, *X*. *vitians*, and *X*. *taraxaci*, could only be distinguished by their host range and were thus transferred as pathovars of the polytypic species *X*. *campestris* (Young et al., 1978).

Based on DNA–DNA hybridization (DDH) (Palleroni & Bradbury, 1993; Vauterin et al., 1995), these five *X*. *campestris* pathovars were classified as pathovars of the new species *X*. *hortorum* (Figure 1). The pathotype strain CFBP 5858^T^ (= LMG 733^T^ = NCPPB 939^T^) of *X*. *hortorum* pv. *hederae* was designated as the species’ type strain. The taxonomical status of “*X*. *hortorum* pv. *vitians*” was unclear, and two variants were distinguished: the former pathotype strain, which is nonpathogenic on lettuce, was labelled “type A”, while “*X*. *hortorum* pv. *vitians*”, pathogenic on lettuce, was designated as “type B”.

A group of strains causing bacterial spot of tomato and pepper (*Solanum lycopersicum* and *Capsicum annuum*) was originally named “*Pseudomonas gardneri*” (Šutic, 1957). Some years later, it was suggested to be part of genus *Xanthomonas* (Dye, 1966), but it was not formally described as *X. gardneri* until the beginning of the 21st century (Jones et al., 2004). The taxonomical history of the *Xanthomonas* strains causing bacterial spot of tomato and pepper has been thoroughly reviewed (Osdaghi et al., 2021; Potnis et al., 2015).

Strains associated with bacterial bract spot of artichoke (*Cynara scolymus*) were first reported in the 1950s as members of the *Xanthomonas* genus (Ridé, 1956), yet the official species description as *X. cynarae* was only provided in 2000 (Trébaol et al., 2000). Although a few phylogenetic studies demonstrated the high genetic relatedness between *X*. *hortorum*, *X*. *cynarae*, and *X*. *gardneri* (Parkinson et al., 2009; Young et al., 2008), they were only recently formally accepted as the same taxonomic entity (Morinière et al., 2020; Timilsina et al., 2019).

**FIGURE 2:**
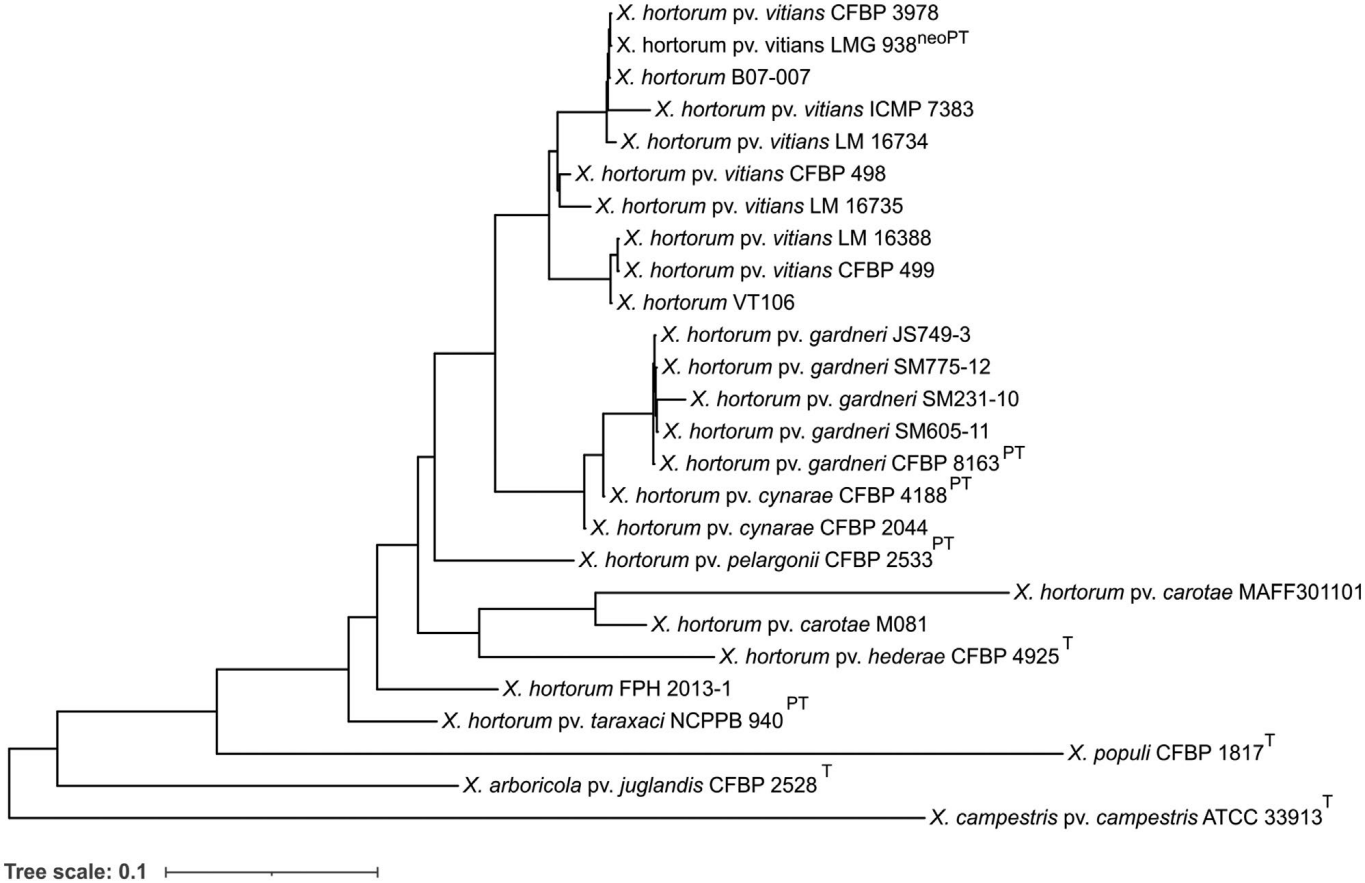
Whole‐genome phylogeny of representative *Xanthomonas hortorum* strains. The tree was constructed using PhyloPhlAn v. 0.40 (Segata et al., 2013) as previously described in Morinière et al. (2020)

Genomic, phenotypic, and pathogenicity analyses were first used to prove the synonymy of *X*. *cynarae* and *X*. *gardneri* and reclassify them as pathovars of *X*. *cynarae* (Timilsina et al., 2019). In that same study, *X*. *hortorum* and *X*. *cynarae* were acknowledged to be paraphyletic species but were kept separate, based on previous wet‐lab DDH results. However, only the type strain of *X*. *hortorum* was included in the 2019 study. A comprehensive analysis revisited the taxonomy of those strains, and included all type, pathotype, or representative strains of *X*. *hortorum* and *X*. *cynarae* (Morinière et al., 2020). Standard genome‐to‐genome comparison parameters, such as average nucleotide identity (ANI), in silico DDH (isDDH), and tetranucleotide frequencies (Tetra), between *X*. *hortorum* and *X*. *cynarae* fell into the transition zone of the species boundary (Morinière et al., 2020), a concept described previously (Richter & Rosselló‐Móra, 2009; Rosselló‐Móra & Amann, 2015). Phylogenetic reconstructions suggested a continuous evolution and diversification of pathovars and phenotypic data did not reveal stable diagnostic traits allowing distinction between *X*. *cynarae* and *X*. *hortorum* strains. *X*. *cynarae* was then suggested to be a later heterotypic synonym of *X*. *hortorum* and both species were combined into an extended *X*. *hortorum* species including seven pathovars (Figure 2): *X*. *hortorum* pvs *hederae*, *pelargonii*, *vitians*, *carotae*, *taraxaci*, *cynarae*, and *gardneri* (Morinière et al., 2020).

## 3 HOST RANGE

Making a distinction between natural and experimental hosts of plant‐pathogenic bacteria is important to better understand the extent of their host range (Bull & Koike, 2015). The natural host range of a pathogen consists of naturally infected plants (i.e., in nonexperimental settings), and is the criterion for pathovar identification and classification (Dye et al., 1980). The experimental host range includes plants that show symptoms after artificial inoculation. Its scope depends on the choice of plant species and of inoculation procedures. The experimental host range provides invaluable information on the pathogen's potential to adapt to new host plants (Jacques et al., 2016).

Each *X*. *hortorum* pathovar has its own natural host range and the experimental host ranges of multiple pathovars have been studied. Additionally, many unassigned strains within *X*. *hortorum* have also been isolated from multiple different plants (e.g., wheat, peony, and hydrangea). Most of the reported natural hosts of *X*. *hortorum* belong to the Geraniaceae, Araliaceae, and Asteraceae families, while most of the reported experimental hosts of the pathogen belong to Asteraceae (Table 1). *X*. *hortorum* affects more than 65 plant species in 15 botanical families, as summarized in Table 1.

**TABLE 1:** The natural and experimental host range of *Xanthomonas hortorum* pathovars and unassigned strains

*X. hortorum* pv.	Isolated from^a^			Host range type^b^	Disease^c^	References
	Family	Plant genus	Plant species			
*carotae*	Apiaceae	*Daucus*	*carota*	N	BLB	Kendrick (1934); Myung et al. (2014); du Toit et al. (2014)
*cynarae*	Asteraceae	*Cynara*	*scolymus*	N	BBS	Trébaol et al. (2000)
	Solanaceae	*Capsicum*	*annuum*	E	NA	Timilsina et al. (2019)
*gardneri*	Asteraceae	*Cynara*	*scolymus*	E	NA	Timilsina et al. (2019)
	Euphorbiaceae	*Euphorbia*	*heterophylla*	N	BS	Araújo et al. (2015)
	Solanaceae	*Solanum*	*lycopersicum*	N	BS	Jones et al. (2004); Quezado‐Duval et al. (2004); Timilsina et al. (2019)
		*Capsicum*	*annuum*	N	BS	Jones et al. (2004); Timilsina et al. (2019)
		*Solanum*	*americanum*	E	BS	Araújo et al. (2015)
		*Nicandra*	*physaloides*	E	BS	Araújo et al. (2015)
	Brassicaceae	*Arabidopsis*	*thaliana*	E	NA	Cândido et al. (2008)
*hederae*	Araliaceae	*Hedera*	*helix*	N	BLS	Arnaud (1920); Trantas et al. (2016)
			*canariensis*	N	BLS	Suzuki et al. (2002)
			*nepalensis* (var. *sinensis*)	N	BLS	Zhang et al. (2015)
			*rhombea*	E	NA	Suzuki et al. (2002)
			*colchica*	E	NA	Leyns et al. (1984)
		*Schefflera*	*atinophylla*	N	BLS	Chase (1984); Norman et al. (1999); Tolba (2017)
			*arboricola*	N	BLS	Chase (1984); Norman et al. (1999)
		*Fatsia*	*japonica*	N	BLS	Chase (1984)
		*Polyscias*	spp.	N	BLS	Norman et al. (1999)
		*Plerandra*	*elegantissima*	E	NA	Chase (1984)
*pelargonii*	Geraniaceae	*Pelargonium*	*capitatum*	N	BB	Knauss and Tammen (1964)
			*peltatum*	N	BB	Starr et al. (1955)
			*quercifolium*	N	BB	Knauss and Tammen (1964)
			*radens*	N	BB	Knauss and Tammen (1964)
			*scandens*	N	BB	Knauss and Tammen (1964)
			*zonale*	N	BB	Leyns et al. (1984)
			× *domesticum*	N	BB	Stapp (1958)
			× *fragrans*	N	BB	Knauss and Tammen (1964)
			× *hortorum*	N	BB	Starr et al. (1955)
			× *ignescens*	N	BB	Knauss and Tammen (1964)
	
		*Geranium*	*maculatum*	N	BB	Stapp (1958)
			*pratense*	N	BB	Starr et al. (1955)
			*sanguineum*	N	BB	Starr et al. (1955)
	*sylvaticum*		N	BB	Stapp (1958)
Euphorbiaceae	*Euphorbia*	*pulcherrima*	E	NA	Rockey et al. (2015)
*taraxaci*		*Taraxacum*	*kok‐saghyz*	N	BLS	Niederhauser (1943)
*vitians*	Asteraceae	*Lactuca*	*sativa*	N	BLS	Brown (1918); Morinière et al. (2020)
			*serriola*	N	BLS	Toussaint et al. (2012); Morinière et al. (2020)
			*biennis*	E	NA	Toussaint et al. (2012)
		*Taraxacum*	*officinale*	E	NA	Toussaint et al. (2012); Morinière et al. (2020)
		*Sonchus*	*oleraceus*	E	NA	Toussaint et al. (2012)
			*asper*	E	NA	Toussaint et al. (2012)
		*Artemisia*	*biennis*	E	NA	Toussaint et al. (2012)
		*Matricaria*	*discoidea*	E	NA	Toussaint et al. (2012)
		*Arctium*	*minus*	E	NA	Toussaint et al. (2012)
		*Gnaphalium*	*uliginosum*	E	NA	Toussaint et al. (2012)
		*Ambrosia*	*artemisiifolia*	E	NA	Toussaint et al. (2012)
		*Galinsoga*	*quadriradiata*	E	NA	Toussaint et al. (2012)
		*Senecio*	*vulgaris*	E	NA	Toussaint et al. (2012)
	Solanaceae	*Nicotiana*	*tabacum*	E	NA	Toussaint et al. (2012)
		*Solanum*	*lycopersicum*	E/N?	BLS	Sahin et al. (2003); Al‐Saleh et al. (2011); Morinière et al. (2020)
		*Capsicum*	*annuum*	E	NA	Sahin et al. (2003); Al‐Saleh et al. (2011)
“*nigromaculans*”		*Arctium*	*lappa*	N	BLS	Parkinson et al. (2009); Dehghan‐Niri and Rahimian (2016)
Unassigned	Asteraceae	*Artemisia*	*annua*	N	BLS	Ssekiwoko et al. (2009)
		*Cichorium*	*intybus*	N	BLS	Zacaroni et al. (2012)
		*Calendula*	*officinalis*	NA	NA	Parkinson et al. (2009)
	Lamiaceae	*Lavandula*	*dentata*	N	BLS	Koike et al. (1995)
			*angustifolia*	N	BLS	Koike et al. (1995); Roberts and Parkinson (2014)
			× *intermedia*	N	BLS	Rotondo et al. (2020)
			× *ginginsii*	E	NA	Rotondo et al. (2020)
	Oleaceae	*Olea*	*europaea*	NA	NA	Young et al. (2010)
	Primulaceae	*Primula*	*vulgaris*	N	BLS	Nejad et al. (2012)
	Hydrangeaceae	*Hydrangea*	*quercifolia*	N	BLS	Cottyn et al. (2021); Uddin et al. (1996)
			*arborescens*	N	BLS	Cottyn et al. (2021)
	Paeoniaceae	*Paeonia*	spp.	N	BB	Oliver et al. (2012); Klass et al. (2019)
	Poaceae	*Triticum*	sp.	E	NA	Egorova et al. (2014)
	Poaceae	*Hordeum*	*vulgare*	E	NA	Egorova et al. (2014)
	Poaceae	*Secale*	*cereale*	E	NA	Egorova et al. (2014)
	Poaceae	*Avena*	*sativa*	E	NA	Egorova et al. (2014)
	Lauraceae	*Persea*	*americana*	NA	NA	Parkinson et al. (2009)

^a^ To ensure consistent botanical taxonomy, plant species nomenclature was checked on the World Flora Online database (WFO, 2021).

^b^ N, natural host; E, experimental host; NA, not applicable.

^c^ Disease type is only mentioned in the event of a natural host. BLS, bacterial leaf spot; BBS, bacterial bract spot; BLB, bacterial leaf blight; BS, bacterial spot; BB, bacterial blight; NA, not applicable.

*X. hortorum* pv. *hederae* is primarily known as a pathogen of English ivy (*Hedera helix*) (Arnaud, 1920; Trantas et al., 2016), but has also been isolated from diseased plants belonging to other *Hedera* species (Table 1). Some *X*. *hortorum* pv. *hederae* strains are pathogenic on several other plants of the Araliaceae family (e.g., *Schefflera* spp.) in natural ecosystems (Table 1). The experimental host range of *X*. *hortorum* pv. *hederae* includes false aralia (*Plerandra elegantissima*), Japanese ivy (*Hedera rhombea*) (Suzuki et al., 2002), and Persian ivy (*Hedera colchica*) (Leyns et al., 1984), but *X*. *hortorum* strains have not been reported on those plants in natural conditions. Another *X*. *hortorum* pathovar, pv. *pelargonii*, naturally occurs on a wide range of plant species from the genera *Geranium* and *Pelargonium* in the Geraniaceae family (Table 1). Some strains of *X*. *hortorum* pv. *pelargonii* cause mild symptoms on poinsettia (*Euphorbia pulcherrima*) in experimental conditions (Rockey et al., 2015).

*X. hortorum* pv. *vitians* is a pathogen of cultivated lettuce (*Lactuca sativa*) and probably infects its closest wild relative, the prickly lettuce (*Lactuca serriola*) (Morinière et al., 2020; Toussaint et al., 2012). This pathovar can be pathogenic on diverse weeds from the Asteraceae family (Table 1) (Toussaint et al., 2012). In greenhouse infection tests, several strains were weakly pathogenic on tomato and two pepper cultivars (*C*. *annuum* ‘Marengo’, a sweet pepper, and *C*. *annuum* ‘Cayenne Long Slim’, a cayenne pepper) (Al‐Saleh et al., 2011; Morinière et al., 2020; Sahin et al., 2003).

To our knowledge the only known hosts of *X*. *hortorum* pvs *carotae* and *taraxaci* are their respective initial hosts of isolation. *X*. *hortorum* pv. *carotae* is pathogenic on wild carrot (*Daucus carota*) and its cultivated subspecies (*D*. *carota* subsp. *sativus*) (Kendrick, 1934; Myung et al., 2014; Temple et al., 2013), while Russian dandelion (*Taraxacum kok‐saghyz*) is the only reported host of *X*. *hortorum* pv. *taraxaci* (Niederhauser, 1943) (Table 1).

The only recorded natural host of *X*. *hortorum* pv. *cynarae* is artichoke (*Cynara scolymus*) (Trébaol et al., 2000) and the pathogen also caused leaf spot symptoms in infiltrated *C*. *annuum* pepper leaves (Timilsina et al., 2019) (Table 1). *X*. *hortorum* pv. *gardneri* is one of the four xanthomonads responsible for bacterial spot of tomato and pepper, alongside *X*. *euvesicatoria* pv. *euvesicatoria*, *X*. *euvesicatoria* pv. *perforans*, and *X*. *vesicatoria* (Jones et al., 2004; Osdaghi et al., 2021; Potnis et al., 2015). *X*. *hortorum* pv. *gardneri* strains were isolated from spot symptoms on tomato and pepper (Jones et al., 2004), as well as the weed plant *Euphorbia heterophylla* (Araújo et al., 2015). Some *X*. *hortorum* pv. *gardneri* strains are pathogenic on tomato or pepper, while other strains are pathogenic on both (Potnis et al., 2015). Moreover, greenhouse inoculations with *X*. *hortorum* pv. *gardneri* resulted in limited necrosis on artichoke leaves (Timilsina et al., 2019), in chlorotic spots on *Arabidopsis thaliana* (Cândido et al., 2008), and in leaf lesions on American black nightshade (*Solanum americanum*) and apple of Peru (*Nicandra physaloides*) (Araújo et al., 2015).

Several unclassified *X*. *hortorum* strains cause disease on other plant species such as peony (*Paeonia* spp.) (Klass et al., 2019; Oliver et al., 2012) and sweet wormwood (*Artemisia annua*) (Ssekiwoko et al., 2009) (Table 1). Strains causing an unknown disease of lavender (*Lavandula dentata*, *L. angustifolia*, and *L*. × *intermedia*) were first identified as *X. campestris* (Koike et al., 1995), but reclassified as *X*. *hortorum* based on sequence data (Roberts & Parkinson, 2014; Rotondo et al., 2020). Strains reported as closely related to *X*. *hortorum* are sometimes unavailable in public or private strain collections, as is the case for angular leaf spot disease of oak‐leaf hydrangea (*Hydrangea quercifolia*) observed in Georgia, USA (Uddin et al., 1996). Recently, similar strains were reported from leaf spot symptoms on hydrangea in Flemish (Belgium) nurseries (Cottyn et al., 2021). Greater burdock (*Arctium lappa*) is also likely to be a natural host of some unassigned *X*. *hortorum* strains (Dehghan‐Niri & Rahimian, 2016).

Other studies suggesting that some strains belong to *X*. *hortorum* have not addressed Koch's postulates. As such, it is unclear whether those strains belong to the species. For example, many *X*. *hortorum* strains were isolated from seed lots of several Poaceae plants in Russia (e.g., wheat, *Triticum* sp.; barley, *Hordeum vulgare*; rye, *Secale cereale*; and oat, *Avena sativa*) (Table 1). Infiltration of bacterial suspension through leaves induced vascular or local necrotic lesions in the corresponding Poaceae species (Egorova, 2015; Egorova et al., 2014). Two multilocus sequence analysis (MLSA) studies reported that strains belonging to *X*. *hortorum* have been isolated from diseased olive (*Olea europaea*) (Young et al., 2010), avocado (*Persea americana*), and pot marigold (*Calendula officinalis*) (Parkinson et al., 2009) (Table 1).

## 4 DISEASE SYMPTOMS

**FIGURE 3:**
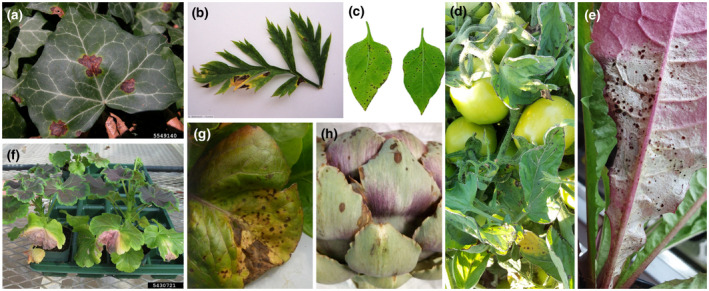
*Xanthomonas hortorum* pathovars on various hosts. (a) English ivy leaf infected by *X*. *hortorum* pv. *hederae*. Courtesy of Forestry Images and the Penn State Department of Plant Pathology & Environmental Microbiology Archives. (b) *X. hortorum* pv. *carotae* symptoms on a carrot leaf. Photograph courtesy of E‐phytia and Benoît Mériaux. (c) *X. hortorum* pv. *gardneri* symptoms on pepper (cv. Early Carl Wonder) leaves, 14 days postinoculation (dpi) with *X*. *hortorum* pv. *gardneri* Xg965. Photograph provided by Neha Potnis. (d) Field infection of tomato plant by *X*. *hortorum* pv. *gardneri*. Photograph provided by Eduardo Bernal. (e) Diseased dandelion leaf 12 dpi after inoculation with *X*. *hortorum* pv. *taraxaci* LM 16389 (= CFBP 8644). Photograph provided by Lucas Morinière. (f) *X*. *hortorum* pv. *pelargonii* on geranium (*Pelargonium* spp.). Photograph courtesy of Forestry Images and Nancy Gregory (University of Delaware). (g) Close‐up of field infection of a lettuce leaf by *X*. *hortorum* pv. *vitians*. Photograph provided by Lucas Morinière. (h) Infection of artichoke head by *X*. *hortorum* pv. *cynarae*. Photograph courtesy of Johan Van Vaerenbergh

The pathovars of *X. hortorum* can cause bacterial spot and/or bacterial blight on numerous plant species. *X. hortorum* pvs *hederae*, *taraxaci*, and *vitians* cause bacterial leaf spot on ivy (Figure 3a), dandelion (Figure 3e), and lettuce (Figure 3g), respectively. *X*. *hortorum* pvs *carotae* and *pelargonii* cause bacterial blight on carrot (Figure 3b) and geranium (Figure 3f), respectively. The symptoms of *X*. *hortorum* pv. *gardneri* can be observed on tomato (Figure 3c) and/or pepper (Figure 3d), depending on the strains, while *X*. *hortorum* pv. *cynarae* causes bacterial spot on artichoke bracts (Figure 3h). The disease symptoms caused by all these pathogens share common characteristics but also have some subtle differences.

Diseases caused by *X*. *hortorum* pathovars are characterized by round, water‐soaked lesions on the abaxial surface of leaves (capitulum artichoke bracts, in the case of *X*. *hortorum* pv. *cynarae*) and are usually the first symptoms observed (Norman et al., 1999; Potnis et al., 2015; Pruvost et al., 2010; Ridé, 1956; Rockey et al., 2015; Schornack et al., 2008; Trébaol et al., 2000). These small water‐soaked leaf spots rapidly expand to form angular necrotic lesions.

The presence of a chlorotic halo around spots or lesions is pathovar‐ or plant/cultivar‐dependent. For example, a chlorotic halo is present around lesions caused by *X*. *hortorum* pvs *pelargonii*, *hederae*, and *taraxaci*, but its presence varies in angular leaf spot caused by *X*. *hortorum* pvs *carotae*, *vitians*, and *gardneri* (Daughtrey & Wick, 1995; Gilbertson, 2002; Myung et al., 2014; Nameth et al., 1999; Pruvost et al., 2010). In advanced infection stages, lesions and spots usually turn dark in colour (brown to black) on plant parts affected by *X*. *hortorum* pvs *pelargonii*, *hederae*, *carotae*, *gardneri*, and *vitians*. They can also coalesce (e.g., in the presence of *X*. *hortorum* pvs *hederae*, *gardneri*, and *vitians*), giving a papery appearance to leaves affected by *X*. *hortorum* pv. *vitians* (Bull & Koike, 2005). In final infection stages, leaves usually harden and dry and, in the case of leaves affected by *X*. *hortorum* pv. *hederae*, a red‐purple margin might appear on their upper surface (Suzuki et al., 2002).

Some very particular leaf symptoms are associated with certain *X*. *hortorum* pathovars. For example, *X*. *hortorum* pv. *pelargonii* can cause leaf margin wilting and V‐shaped necrotic areas, depending on the plant species and cultivar (Daughtrey & Wick, 1995). The affected areas eventually drop off, and black stem rot occurs in case of a systemic infection. When the infection expands to the roots, it results in overall wilt and gradual plant death, but no decay or soft rot is observed (Daughtrey & Benson, 2005; Manulis et al., 1994).

Furthermore, leaves are not the only plant parts affected by *X*. *hortorum*. *X*. *hortorum* pv. *gardneri* affects tomato fruits, on which it causes characteristic star‐shaped lesions with a raised, scabby appearance (Potnis et al., 2015). On unripe tomato fruits, symptoms look like water‐soaked or slightly raised pale‐green spots, sometimes surrounded by greenish‐white halos. On tomato sepals, symptoms consist of brown lesions, which can turn necrotic; stem lesions are narrow, elongated, and raised (Potnis et al., 2015). *X*. *hortorum* pv. *hederae* occasionally affects stems and petioles (Suzuki et al., 2002), and *X*. *hortorum* pv. *carotae* causes disease on petioles, peduncles, stems, flowers, and leaflets (Gilbertson, 2002). Lesions caused by *X*. *hortorum* pvs *carotae* and *vitians* can be V‐shaped (Gilbertson, 2002; Sahin, 1997; Scott & Dung, 2020; du Toit et al., 2014).

## 5 GEOGRAPHIC DISTRIBUTION AND IMPORTANCE

**FIGURE 4:**
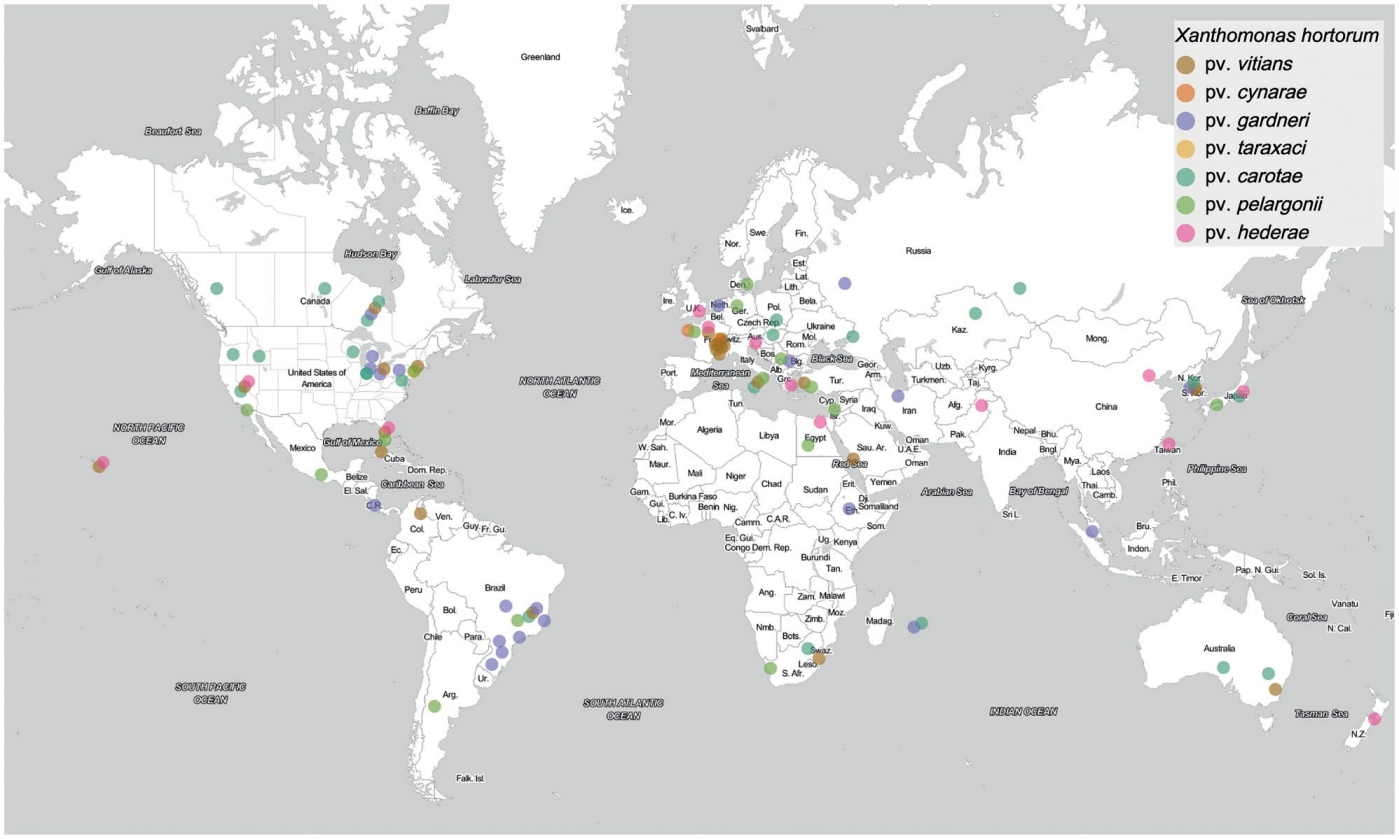
Distribution of the seven *Xanthomonas hortorum* pathovars. Map from the ggmap R package (Kahle et al., 2019) and data adapted from the European and Mediterranean Plant Protection Organization (EPPO). Location is an approximation based on literature available

*X. hortorum* includes a pathovar causing the most devastating bacterial disease of geranium (pv. *pelargonii*) (Manulis et al., 1994; Munnecke, 1954), an internationally regulated seedborne pathovar affecting carrot (pv. *carotae*) (Scott & Dung, 2020), and a pathovar reported in most lettuce‐growing areas (pv. *vitians*) (Sahin, 1997). Furthermore, *X*. *hortorum* pv. *gardneri*, in addition to three other *Xanthomonas* spp., is a major pathogen on tomato and/or pepper (Jones et al., 2004; Osdaghi et al., 2021; Potnis et al., 2015). Diseases caused by *X*. *hortorum* pathovars have been reported in more than 40 countries across all continents except Antarctica (Figure 4), either as one‐time reports or as frequent reoccurrences. One‐time reports do not necessarily mean that the diseases are not recurring or currently present. Examples of frequent reoccurrences include *X*. *hortorum* pv. *carotae* in Canada and the USA (EPPO, 2021), *X*. *hortorum* pv. *vitians* in Canada and France (Morinière et al., 2020; Toussaint, 1999; Toussaint et al., 2012), and *X*. *hortorum* pv. *gardneri* in the USA and Brazil (Araújo et al., 2012, 2015, 2017; Potnis et al., 2015; Quezado‐Duval et al., 2004), the second and sixth largest tomato producers in 2019, respectively, by gross production value (FAOSTAT, 2021).

Data on the economic impact of *X*. *hortorum* is not available for all its pathovars, as there are no reports documenting the cost of the damage caused by *X*. *hortorum* pvs *cynarae*, *taraxaci*, and *hederae*. When available, economic impact reports are not recent but are nonetheless informative about the scope of the importance of *X*. *hortorum*. For example, bacterial blight of geranium caused by *X*. *hortorum* pv. *pelargonii* is the most devastating bacterial pathogen of geranium and can lead to total geranium loss when environmental conditions are most favourable to this pathogen (Balaž et al., 2016; Manulis et al., 1994; Munnecke, 1954; Nameth et al., 1999). Most carrot seed growers in the Pacific Northwest region of the USA, an important region for US carrot seed production, consider *X*. *hortorum* pv. *carotae* detrimental to seed quality (Dr Jeremiah Dung, The Oregon State University, March 2021, personal communication). In Montreal, Canada, losses due to *X*. *hortorum* pv. *vitians* have led to complete destruction of lettuce fields (Toussaint, 1999). In Florida, USA, the pathovar caused an estimated loss of $4 million from the early to mid‐1990s (Robinson et al., 2006), and also caused substantial economic losses in California and Ohio, USA (Carisse et al., 2000; Sahin, 1997). Losses due to *X*. *hortorum* pv. *gardneri* were estimated to cost the Midwestern US tomato‐processing industry $7–8 million (Ma, 2015; Ma et al., 2011).

*X. hortorum* pv. *gardneri* is one of the four xanthomonads causing bacterial spot of tomato and pepper, and multiple reports have studied population structure shifts of those four species, especially in the USA and Brazil (Araújo et al., 2015, 2017; Egel et al., 2018; Pereira et al., 2011). In the USA, most early reported incidences of bacterial spot disease on tomato and pepper were caused by *X*. *euvesicatoria* pv. *euvesicatoria* but a population shift to *X*. *hortorum* pv. *gardneri* has been reported in published work (Egel et al., 2018; Ma, 2015; Ma et al., 2011) and personal communications (Dr Francesca Rotondo and Dr Sally A. Miller, The Ohio State University, March 2021, personal communication). However, recent surveys for tomato and pepper bacterial spot in Brazil have shown a limited presence of *X*. *hortorum* pv. gardneri (Araújo et al., 2017).

## 6 EPIDEMIOLOGY

In general, *X*. *hortorum* pathovars thrive in warm, wet, and humid environments in fields and greenhouses (Dye, 1967; Gardner & Kendrick, 1921; Kendrick, 1934; Manulis et al., 1994; Strider, 1985; du Toit et al., 2005; Toussaint, 1999). During inoculation trials, *X*. *hortorum* pv. *gardneri* had a higher virulence at 20°C when compared to other bacterial spot pathogens of tomato and pepper (Araújo et al., 2011), and was more prevalent than other bacterial spot xanthomonads at higher altitudes (Araújo et al., 2017). Furthermore, *X*. *hortorum* pv. *vitians* has an optimal infection temperature of around 23°C (Robinson et al., 2006).

The pathovars colonize the plants through natural openings (e.g., hydathodes, stomata) or wounds (Bernal & Francis, 2021; Dougherty et al., 1974; Ridé, 1956; Schwartz et al., 2017). After gaining entry, they infect the plant vascular system (Barak et al., 2002; Munnecke, 1954). Mesophyll colonization is also possible for *X*. *hortorum* pvs *pelargonii* (Barel et al., 2015) and *vitians* (authors’ unpublished data). Infections of *X*. *hortorum* pvs *pelargonii* and *vitians* can sometimes be symptomless (Barak et al., 2002; McPherson & Preece, 1978).

The two primary sources of inoculum of *X*. *hortorum* pathovars are seeds and cuttings, although they can be disseminated through other means as well (e.g., insects, rain, and irrigation water) and can survive on weeds, crop debris, or in soils. Seed is a main source of inoculum for bacterial spot and blight caused by *X*. *hortorum* pvs *carotae*, *gardneri*, and *vitians* (Barak et al., 2001, 2002; Kendrick, 1934; Kuan, 1985; Mtui et al., 2010; Sahin & Miller, 1997; du Toit et al., 2005, 2014). Contaminated seed, stecklings, or seedlings may initiate an epidemic in grower fields (McDonald & Linde, 2002; du Toit et al., 2005), which could result in a nonnormal pathogen distribution, as observed for *X*. *hortorum* pv. *carotae* populations (Scott & Dung, 2020). This can pose a challenge to the development of detection methods and durable resistant cultivars.

*X. hortorum* pvs *hederae* and *pelargonii* are mainly transmitted by infected cuttings (Chittaranjan & De Boer, 1997; Norman et al., 1999) because flowers such as geraniums are commonly vegetatively propagated by cuttings. Historically, propagating facilities were inadvertently responsible for distributing infected symptomless plant material (Nameth et al., 1999).

Crop residues can allow *X*. *hortorum* pvs *carotae* and *vitians* to overwinter for several months or until the following growing season (Christianson et al., 2015; Sahin et al., 2003). *X*. *hortorum* pv. *carotae* can persist in infected carrot foliage on soil for up to a year (Gilbertson, 2002). *X*. *hortorum* pv. *vitians* can survive in crop debris for up to 1 month, in both summer and winter months (Barak et al., 2001; Fayette et al., 2018). *X*. *hortorum* pvs *vitians* and *gardneri* can survive epiphytically or infect weeds, respectively (Araújo et al., 2015; Barak et al., 2001; Fayette et al., 2018). Soil or crop debris also act as an important inoculum source for *X. hortorum* pv. *carotae*, where it can survive for up to 3 months (Kendrick, 1934), and for pv. *pelargonii*, which can survive in soils for up to a year (Gilbertson, 2002). Survival in weeds, plant residues, and soils can serve as a secondary inoculum source in the presence of favourable hosts and environmental conditions (Gitaitis & Walcott, 2007). If bacterial populations are high, they can re‐emerge from inside the plant tissue and serve as a secondary inoculum on the plant itself or on nearby hosts.

*X. hortorum* pvs *gardneri*, *carotae*, and *vitians* are also disseminated by wind or rain, or mechanically transferred during planting and cultivation (Potnis et al., 2015; du Toit et al., 2005). *X*. *hortorum* pv. *carotae* has been observed in aerosolized debris generated by carrot seed threshers during field operations (du Toit et al., 2005). *X*. *hortorum* pv. *pelargonii* can be transmitted by greenhouse whiteflies (*Trialeurodes vaporariorum*) (Bugbee & Anderson, 1963), and insects were noted to be vectors for *X*. *hortorum* pv. *carotae* but no details (insect genus or species) were given (Gilbertson, 2002).

## 7 IDENTIFICATION AND DETECTION

Visual symptom assessment is the first step to detect a suspected *X*. *hortorum* infection and subsequent identification is based on pathogen isolation. *X*. *hortorum* strains are readily isolated from infected plant tissue using serial dilution plating. Growth media used can be nonselective (e.g., nutrient agar, sucrose peptone, or yeast‐dextrose‐calcium carbonate [YDC] agar) or semiselective (Saddler & Bradbury, 2015). Irrespective of medium type, *X*. *hortorum* colonies are yellow, mucoid, and convex (Saddler & Bradbury, 2015).

Phenotypic profiles of this species, analysed using phenotype microarrays (e.g., Biolog, OMNILOG), remain too variable to provide an accurate identification at the species level (Akhtar & Aslam, 1990; Bouzar et al., 1999; Mirik et al., 2018; Morinière et al., 2020; Myung et al., 2010; Stoyanova et al., 2014; Trébaol et al., 2000; Uddin et al., 1996). Pathovars cannot be distinguished from one another by using such phenotypic profiling as no stable, discriminative traits exist (Morinière et al., 2020; Trébaol et al., 2000). Even though pathovar classification depends on host pathogenicity (see *Taxonomy* *update*), the identification of *X*. *hortorum* pathovars should not solely rely on the host range. Indeed, some strains of this species can naturally infect hosts other than their original host of isolation (see *Host range*).

SDS‐PAGE protein profiling and later DDHs (Stefani et al., 1994; Vauterin et al., 1991, 1995) were used to identify *X*. *hortorum* pv. *vitians* “type B”, revealing the existence of aberrant strains (Table 2). Even though fatty acid profiling did not provide identification among pathovars and often remains inaccurate at the species level (Barak & Gilbertson, 2003; Mirik et al., 2018; Sahin et al., 2003; Ssekiwoko et al., 2009; Uddin et al., 1996), it still distinguished between *X*. *hortorum* pv. *vitians* “type B” and the unusual isolates (Sahin et al., 2003). Furthermore, a panel of 16 xanthomonad‐specific monoclonal antibodies (Table 2), used in enzyme‐linked immunosorbent assays (ELISAs), distinguished two serovars of *X*. *hortorum* pv. *vitians* isolates (Sahin et al., 2003).

**TABLE 2:** Non‐DNA and DNA‐based identification methods for *Xanthomonas hortorum* pathovars. The detection targets, taxonomical level of detection, and primer sequence availability, when applicable, are also reported

Detection				Targeted pathovar	Specific for the targeted pathovar (antibody, primer, or probe name)^b^	Reference(s)	Comments^c^
method	Type	Target(s)	Taxonomical level^a^				
ELISA	Non‐DNA	Polyclonal antibodies	Pathovar	pv. *pelargonii*	Yes	Balaž et al. (2016)	Commercial ELISA kit
ELISA	Non‐DNA	Monoclonal antibodies	*Xanthomonas* species	pv. *pelargonii*	Yes (MAb Xpel‐1)	Benedict et al. (1990)	NA
ELISA	Non‐DNA	Monoclonal antibodies	Pathovar	pv. *pelargonii*	Yes (McAb 2H5)	Chittaranjan and De Boer (1997)	NA
ELISA	Non‐DNA	Monoclonal antibodies	*Xanthomonas* species/pathovars	pv. *vitians*	No	Sahin et al. (2003)	Pattern‐based discrimination
SDS‐PAGE	Non‐DNA	Various proteins	*Xanthomonas* species/pathovars	various: pvs *vitians* (cluster 7d), *hederae* (cluster 7e), *pelargonii* (cluster 12)	No	Vauterin et al. (1991)	Pattern‐based discrimination
SDS‐PAGE	Non‐DNA	Various proteins	*Xanthomonas* species/pathovars	pv. *vitians*	No	Stefani et al. (1994)	Pattern‐based discrimination
SDS‐PAGE	Non‐DNA	Various proteins	Pathovar	pv. *gardneri*	No	Quezado‐Duval et al. (2004)	Pattern‐based discrimination
DDH	DNA	DNA homology groups	*Xanthomonas* species/pathovars	various: pvs *pelargonii*, *hederae*, *vitians* (group 2)	No	Vauterin et al. (1995)	Clustering‐based discrimination
MLSA/MLST	DNA	Various housekeeping genes, including *gyrB*	*Xanthomonas* species	various pvs	No	Parkinson et al. (2007); Young et al. (2008); Parkinson et al. (2009)	Clustering‐based discrimination
MLSA/MLST	DNA	Various housekeeping genes, including *gyrB*	Pathovar	pv. *vitians*	No	Fayette et al. (2016)	Clustering‐based discrimination
PCR	DNA	RAPD fragments	Pathovar	pv. *carotae*	Yes (3S/3SR and 9B/9BR)	Meng et al. (2004)	D.L.: 22 fg (3S) and 2 pg (9B)
PCR	DNA	CDS or intergenic regions	Pathovar	pv. *carotae*	Yes (XhcPP02, PP03, PP04, and PP05)	Kimbrel et al. (2011)	NA
PCR	DNA	Based on the target of XhcPP02 (Kimbrel et al., 2011)	Pathovar	pv. *carotae*	Yes (Xhc‐q2)	Temple et al. (2013)	NA
PCR	DNA	AFLP fragments	BSX	pv. *gardneri*	Yes (Bs‐XgF/Bs‐XgR)	Koenraadt et al. (2009); Pereira et al. (2011)	NA
PCR	DNA	1.2 kb DNA‐fragment	Pathovar	pv. *pelargonii*	Yes	Manulis et al. (1994); Chittaranjan and De Boer (1997)	NA
PCR	DNA	ERIC, REP regions	Pathovar	pv. *pelargonii*	No	Sulzinski et al. (1995)	Pattern‐based discrimination
PCR	DNA	ERIC fragment	Pathovar	pv. *pelargonii*	Yes (XcpMl/XcpM2)	Sulzinski et al. (1996); Sulzinski et al. (1997); Sulzinski et al. (1998); Sulzinski (2001)	NA
PCR	DNA	RAPD fragments	Pathovar	pv. *vitians*	Yes (B162)	Barak et al. (2001)	NA
PCR	DNA	BOXA, ERIC, REP, and 16S‐23S rDNA regions	*Xanthomonas* species/pathovars	pv. *vitians*	No	Sahin et al. (2003)	Pattern‐based discrimination
PCR	DNA	SNP‐based	Pathovar	pv. *vitians*	No	Hébert et al. (2021)	*C*_t_ values
Multiplex PCR	DNA	ERIC fragment	*X. hortorum* pv. *pelargonii* and *Ralstonia solanacearum*	pv. *pelargonii*	Yes (DG1/DG2)	Glick et al. (2002)	NA
PCR and multiplex PCR	DNA	AFLP fragments	BSX	pv. *gardneri*	Yes (Bs‐XgF/Bs‐XgR)	Araújo et al. (2012)	D.L.: DNA: 50 pg/µl; bacterial suspension: 5 × 10^4^ cfu/ml (100 bacterial cells per reaction).
Real‐time PCR	DNA	ERIC fragment	Pathovar	pv. *pelargonii*	Yes (XhqF/XhqR)	Farahani and Taghavi (2016)	D.L.: 200 fg
Multiplex real‐time PCR	DNA	*lepA*	BSX +*Clavibacter* *michiganensis* subsp. *michiganensis*, *Pseudomonas syringae* pv. *tomato*	pv. *gardneri*	No	Peňázová et al. (2020)	Cy5 was used for multiplex PCR that targeted BSX together
Multiplex real‐time PCR	DNA	*hrpB7* primers and probes	BSX	pv. *gardneri*	Yes, in combination with probe (FP2/RP2)	Strayer, Jeyaprakash, et al. (2016)	D.L.: 5 × 10^5^ cfu/ml
LAMP	DNA	Based on PCR product of 9B primer set (Meng et al., 2004)	Pathovar	pv. *carotae*	Yes (Lace primer set)	Temple and Johnson (2009); Temple et al. (2013)	NA
LAMP	DNA	*hrpB6*	Pathovar	pv. *gardneri*	Yes	Stehlíková et al. (2020)	D.L.: 1 pg/µl
RPA	DNA	*hrpB6*	Pathovar	pv. *gardneri*	No (XGF/XGR)	Strayer‐Scherer et al. (2019)	D.L.: 5 × 10^6^ cfu/ml; amplified *X. hortorum* pv. *cynarae* CFBP 2044

^a^ BSX, the *Xanthomonas species* causing bacterial spot of tomato and pepper: *X. hortorum* pv. *gardneri*, *X. vesicatoria*, and *X. euvesicatoria* pvs *euvesicatoria* and *perforans*.

^b^ Primer sequences are available in all the DNA‐based detection methods.

^c^ D.L., reported detection limits; NA, not available.

Antibodies were used to detect *X*. *hortorum* pv. *pelargonii*. Pathovar‐specific monoclonal antibodies (Benedict et al., 1990; Chittaranjan & De Boer, 1997) and polyclonal antibodies (Balaž et al., 2016; Mirik et al., 2018) were successfully used for serological identification of this pathogen (Table 2), using commercial double‐antibody sandwich ELISA kits (LOEWE Biochemica GmbH and Agdia).

Several DNA‐based molecular assays have been developed over recent decades to identify and detect *X*. *hortorum* strains (Table 2). The available methods are limited to four of the seven pathovars, as diagnostics methods are unavailable for *X*. *hortorum* pvs *hederae*, *taraxaci*, and *cynarae* at the time of writing. Several PCR detection protocols are available to amplify DNA from many *Xanthomonas* species, including one or more *X*. *hortorum* pathovars, by targeting the 16S rRNA gene (Maes, 1993), the *hrp* gene cluster (Leite et al., 1994), or *gumD*, *fyuA,* and the internal transcribed spacer (ITS) (Adriko et al., 2014). However, these general protocols are usually not implemented for the identification and detection of *X*. *hortorum* pathovars. Instead, targeted assays allowing specific detection and identification at the pathovar level are often preferred.

The first targeted detection DNA‐based assays were mostly derived from DNA fingerprint methods, and were used to study the genetic diversity of *X*. *hortorum* pathovars (Barak & Gilbertson, 2003; Hamza et al., 2012; Sahin et al., 2003; Sulzinski, 2001). For example, a PCR for *X*. *hortorum* pv. *carotae* was developed from random amplified polymorphic DNA (RAPD) analysis (Meng et al., 2004). Similarly, diagnostic PCR tests for *X*. *hortorum* pv. *pelargonii* were developed from specific DNA fragments identified by RAPD analysis, enterobacterial repetitive intergenic consensus (ERIC) PCR or repetitive extragenic palindromic (REP) PCR (Chittaranjan & De Boer, 1997; Manulis et al., 1994; Sulzinski, 2001; Sulzinski et al., 1995, 1996, 1997, 1998). A multiplex PCR scheme for the simultaneous detection of *X*. *hortorum* pv. *pelargonii* and members of the *Ralstonia solanacearum* species complex, the second major bacterial pathogen of geranium, was developed from one of the two previously identified ERIC‐PCR fragments (Glick et al., 2002). The same molecular region was used to develop a real‐time quantitative PCR (qPCR) assay, allowing quantification of the pathogen (Farahani & Taghavi, 2016).

For *X*. *hortorum* pv. *gardneri*, a marker identified using amplified fragment polymorphism (AFLP) was initially used to design a diagnostic PCR assay (Koenraadt et al., 2009). The assay was later adapted into a multiplex PCR targeting four *Xanthomonas* species associated with tomato bacterial spot (Araújo et al., 2012). A multiplex TaqMan qPCR assay differentiating these four species was based on *hrpB7*, a less conserved gene within the *hrpB* operon (Strayer, Jeyaprakash, et al., 2016). A multiplex qPCR detecting these four *Xanthomonas* species, as well as tomato pathogens *Clavibacter michiganensis* subsp. *michiganensis* and *Pseudomonas syringae* pv. *tomato*, has been recently developed by targeting *lepA* (Peňázová et al., 2020).

Two *X*. *hortorum* pv. *vitians*‐specific primer pairs, 9308B and B162, were developed from RAPD fragments (Barak et al., 2001), but their specificity to the target varied. Primer pair 9308B failed to amplify isolates recovered from lettuce and weeds around lettuce fields. On the other hand, primer pair B162 successfully detected *X*. *hortorum* pv. *vitians* strains isolated over a 7‐year period (Barak et al., 2001).

Partial gene sequence of *gyrB* offers a sufficient resolution for the identification of xanthomonad isolates at the species level (Parkinson et al., 2007, 2009). MLSA is preferred to single‐gene (e.g., *gyrB*) to outline the precise phylogeny of *X*. *hortorum* (Morinière et al., 2020). However, MLSA schemes are based on different partial gene sequences, (sub)sets of partial genes, and trimming settings, which complicates the analysis by not allowing proper comparison between studies (Catara et al., 2021). The sequencing of the first draft genome of *X*. *hortorum* pv. *carotae* in 2011 allowed the first use of comparative genomics to develop two new diagnostics assays to detect this pathovar (Kimbrel et al., 2011; Temple et al., 2013). The first assay used a TaqMan qPCR, whereas the second relied on loop‐mediated isothermal amplification (LAMP) (Temple et al., 2013). The latter method showed superior performance compared to qPCR because of its robustness in the presence of inhibitors, and its rapidity, versatility, and usefulness in facilities with limited resources (Kimbrel et al., 2011). Both assays were also the first ones to be used as viability assays (i.e., detection of viable bacterial cells) with a xanthomonad, by including a propidium monoazide treatment prior to DNA extraction.

Two other isothermal amplification methods were recently published for the in‐field detection of *X*. *hortorum* pv. *gardneri* (Table 2), with an emphasis on differentiation from the other xanthomonad species responsible for tomato bacterial spot. The first method is based on recombinase polymerase amplification (RPA) and targets *hrcN* (*hrpB*) (Strayer‐Scherer et al., 2019). The second, based on LAMP, targets partial *hrpB* gene sequence (Stehlíková et al., 2020).

## 8 GENOMICS

The genome of *X*. *hortorum* pv. *gardneri* ATCC 19865^PT^ (Potnis et al., 2011) was the first *X*. *hortorum* genome publicly available in the National Center for Biotechnology Information (NCBI) database (accessed March 2021). At the time of writing, 35 *X*. *hortorum* genomes have been deposited in the database, and most (46%) were submitted in 2020 (Table 3). Their completeness varies, and 74% (*n* = 26) are incomplete (i.e., assembled at the scaffold or contig levels; Table 3). Eight of the nine complete *X*. *hortorum* genomes contain at least one plasmid sequence. The average genome size of *X*. *hortorum* is 5.26 Mb (4.92–5.68 Mb). The average G + C content is of 63.6% (63.2%–63.9%), and the average predicted coding sequence (CDS) number is 4260 (Table 3). The average size of *X*. *hortorum* plasmids is 86.90 kb (29.56–224.70 kb), and three plasmids in *X*. *hortorum* pvs *vitians* and *gardneri* genomes are larger than 100 kb (Table 3). The average G + C content of plasmids is 60.30% (58.07%–62.18%), with an average of 94 predicted CDS.

**TABLE 3:** Genome metrics of representative *Xanthomonas hortorum* strains

Organism	Submission year	Strain	GenBank assembly accession	Assembly level	Contigs/scaffolds	Size (Mb)	GC (%)	N_50_ (bp)	CDS^a^	Plasmids (bp)
*X. hortorum*	2017	B07‐007	GCA_002285515.1	Complete	2	5.25	63.6	5,175,249	4241	pB07007 (75,655 bp)
*X. hortorum*	2019	VT 106	GCA_008728175.1	Complete	2	5.15	63.7	5,101,806	4135	pVT106 (44,015 bp)
*X. hortorum*	2020	FPH2013‐1	GCA_011305375.1	Scaffold	70	5.22	63.7	190,176	4187	–
*X. hortorum* pv. *carotae*	2013	M081	GCA_000505565.1	Chromosome	1	5.05	63.7	5,052,399	8446	–
*X. hortorum* pv. *carotae*	2020	MAFF 301101	GCA_015726835.1	Contig	423	5.10	63.7	20,618	3972	–
*X. hortorum* pv. *cynarae*	2018	CFBP 4188^PT^	GCA_002939985.1	Scaffold	102	5.06	63.7	145,505	4060	–
*X. hortorum* pv. *cynarae*	2020	CFBP 2044	GCA_903978235.1	Complete	2	5.12	63.7	5,079,002	8640	CFBP2044_p40 (40,232 bp)
*X. hortorum* pv. *gardneri*	2015	SM234‐10	GCA_001009295.1	Scaffold	179	5.33	63.5	49,361	4337	–
*X. hortorum* pv. *gardneri*	2015	SM605‐11	GCA_001009325.1	Scaffold	158	5.34	63.5	61,942	4327	–
*X. hortorum* pv. *gardneri*	2015	SM775‐12	GCA_001009625.1	Scaffold	176	5.25	63.6	59,240	4244	–
*X. hortorum* pv. *gardneri*	2016	JS749‐3	GCA_001908755.1	Complete	3	5.42	63.5	5,158,913	4373	pJS749‐3.1 (211,336 bp), pJS749‐3.2 (45,952 bp)
*X. hortorum* pv. *gardneri*	2020	CFBP 8163^PT^	GCA_012922265.1	Contig	121	5.15	63.7	125,969	4151	–
*X. hortorum* pv. *hederae*	2018	CFBP 4925^T^	GCA_002940005.1	Scaffold	313	5.32	63.8	42,684	4299	–
*X. hortorum* pv. *pelargonii*	2020	CFBP 2533^PT^	GCA_012922215.1	Contig	94	5.21	63.8	134,256	4176	–
*X. hortorum* pv. *taraxaci*	2020	NCPPB 940^PT^	GCA_903978185.1	Complete	2	5.03	63.8	4,999,567	8628	NCPPB940_p30 (29,567 bp)
*X. hortorum* pv. *vitians*	2020	LM 16735	GCA_012922125.1	Contig	138	5.19	63.7	118,265	4192	–
*X. hortorum* pv. *vitians*	2020	LMG 938^neoPT^	GCA_012922135.1	Contig	119	5.03	63.8	141,718	4036	–
*X. hortorum* pv. *vitians*	2020	LM 16388	GCA_012922175.1	Contig	121	5.07	63.7	115,523	4063	–
*X. hortorum* pv. *vitians*	2020	CFBP 3978	GCA_012922195.1	Contig	131	5.13	63.7	141,718	4137	–
*X. hortorum* pv. *vitians*	2020	CFBP 499	GCA_012922335.1	Contig	132	5.17	63.7	118,955	4161	–
*X. hortorum* pv. *vitians*	2020	LM 16734	GCA_014338485.1	Complete	2	5.27	63.7	5,213,310	4223	pLM16734 (57,250 bp)
*X. hortorum* pv. *vitians*	2020	CFBP 498	GCA_903978195.1	Complete	4	5.68	63.2	5,365,193	4654	CFBP498_p224 (224,704 bp), CFBP498_p47 (47,063 bp), CFBP498_p41 (41,583 bp)
*X. hortorum* pv. *vitians*	2016	ICMP 7383	GCA_001908775.1	Complete	4	5.63	63.3	5,313,102	4511	pICMP7383.1 (203,385 bp), pICMP7383.2 (61,840 bp), pICMP7383.3 (47,122 bp)

^a^ The number of CDS is a direct output from NCBI and three numbers appear to overestimate the actual number.

The essential genome (genes required for growth and survival, irrespective of environmental conditions; Koonin, 2000) of *X*. *hortorum* pv. v*itians* LM 16734 was recently characterized through saturated transposon insertion sequencing (Morinière et al., 2021) and included 370 protein‐coding genes. These genes were mostly associated with critical cellular processes (e.g., translation, energy production, lipid transport), with 355 and 334 of them conserved within *X*. *hortorum* and the *Xanthomonadaceae* family, respectively.

### 8.1 Lipo‐ and exo‐polysaccharides

Lipopolysaccharides (LPSs) are involved in biofilm formation and protecting pathogens from their environment (Corsaro et al., 2001; Newman et al., 2002, 2007). *X*. *hortorum* pv. *gardneri* ATCC 19865^PT^ has a 17.7 kb ancestral‐type LPS gene cluster, like that of *X*. *campestris* pv. *campestris* ATCC 33913^T^ (Potnis et al., 2011). The LPS gene clusters of *X*. *hortorum* pvs *cynarae* CFBP 4188^PT^ and *gardneri* ATCC 19865^PT^ were highly syntenic, but different to that of *X*. *hortorum* pv. *hederae* CFBP 4925^T^ (Timilsina et al., 2019). *X*. *hortorum* pvs *carotae* M081, *cynarae* CFBP 4188^PT^, *gardneri* ATCC 19865^PT^, and *hederae* CFBP 4925^T^ possess *wzm* and *wzt* homologs (Kimbrel et al., 2011; Potnis et al., 2011; Timilsina et al., 2019), involved in the transport of LPS band A in *Pseudomonas aeruginosa* (Rocchetta & Lam, 1997).

Exopolysaccharides (EPSs) are involved in xanthan biogenesis, and they protect xanthomonad pathogens from environmental stress (Kakkar et al., 2015; Kamoun & Kado, 1990; Sutherland, 1993). The EPS gene cluster of *X*. *hortorum* pvs *carotae* and *vitians* is arranged similarly to that of *X*. *campestris* pv. *campestris* and contains all 12 genes from the *gumB*‐*gumM* cluster (Kimbrel et al., 2011; Morinière et al., 2021). Unlike in other *Xanthomonas* species (Katzen et al., 1998; Kim et al., 2008), only the mutations of *gumE*, *gumI*, and *gumJ* were lethal in *X*. *hortorum* pv. *vitians* LM 16734 (Morinière et al., 2021). The presence of a tRNA gene flanking the cluster in some *Xanthomonas* genomes suggests a horizontal transfer acquisition (Lu et al., 2008). However, no evidence of insertion elements was found in the EPS gene cluster of *X*. *hortorum* pvs *carotae* and *vitians* (Kimbrel et al., 2011; Morinière et al., 2021).

### 8.2 Secretion systems

Secretion systems and their effector proteins are crucial determinants of virulence in the *Xanthomonas* genus (Büttner & Bonas, 2010). There are two types of type II secretion system (T2SS) clusters within *Xanthomonas*: the T2SS‐*xps*, directly involved in virulence, and the T2SS‐*xcs*, which has seemingly no direct virulence function (Szczesny et al., 2010). The pathotype strains of *X*. *hortorum* pvs *hederae*, *gardneri*, and *cynarae*, in addition to strain B07‐007, have complete T2SS‐*xps* (*xpsD*‐*xpsN*) and T2SS‐*xcs* (*xcsC*‐*xcsN*) clusters (Alvarez‐Martinez et al., 2021; Timilsina et al., 2020). Unlike T2SS‐*xps*, the T2SS‐*xcs* cluster is not conserved within *Xanthomonas* spp. (Timilsina et al., 2020) but is conserved between the four *X*. *hortorum* strains.

The type III secretion system (T3SS) delivers effector proteins that, in turn, can suppress or trigger plant defence mechanisms (Büttner, 2016; White et al., 2009). The T3SS is found in most *Xanthomonas* strains, including *X*. *hortorum* (Timilsina et al., 2020). The T3SS of *X*. *hortorum* pv. *gardneri* ATCC 19865^PT^ is a mosaic *hrp* cluster, with elements like that of *X*. *campestris* pv. *campestris* ATCC 33913^T^, but also including novel effectors (see *Molecular host–pathogen interactions*) (Potnis et al., 2011). *X*. *hortorum* pv. *carotae* M081 has a complete *hrp* cluster and is predicted to be functional (Kimbrel et al., 2011). Furthermore, a recent study reported that the T3SS *hrp* cluster in *X*. *hortorum* pv. *gardneri* ATCC 19865^PT^, *cynarae* CFBP 4188^PT^, *hederae* CFBP 4925^T^, and *carotae* M081 are similar, with some differences in the two 20 kb regions flanking the cluster (Merda et al., 2017).

The type IV secretion system (T4SS) is involved in protein transfer as well as bacterial conjugation (Guglielmini et al., 2014; Lawley et al., 2003; Llosa et al., 2002). *X*. *hortorum* pv. *gardneri* ATCC 19865^PT^ has two plasmidborne and one chromosomal T4SS clusters (Potnis et al., 2011). The chromosomal cluster of *X*. *hortorum* pv. *gardneri* is complete and similar to that of *X*. *campestris* pv. *campestris* ATCC 33913^T^. One of the two *X*. *hortorum* pv. *gardneri* plasmidborne clusters is 98% and 89% identical to the T4SS clusters of *Burkholderia multivorans* ATCC 17616 and *Acidovorax avenae* subsp. *citrulli* AAC001, respectively. The other plasmidborne cluster is similar to the one found in *X*. *vesicatoria* ATCC 35937^T^ and *X*. *euvesicatoria* pv. *perforans* 91‐118 (Potnis et al., 2011). The presence of a T4SS cluster in *X*. *hortorum* pv. *carotae* M081 was suggested by the detection of *virB* genes scattered over three different contigs but its functionality was inconclusive (Kimbrel et al., 2011).

The type V secretion system (T5SS) is responsible for the secretion of various proteins, including adhesins, which are important for host colonization as they are among the first contact points between pathogen and host (Meuskens et al., 2019). The members of T5SS are autotransporters, with the exception of type 5b, which is formed of two proteins (Guérin et al., 2017). In *Xanthomonas* spp., T5SS clusters belong to categories 5a, 5b, and 5c (Alvarez‐Martinez et al., 2021). *X. hortorum* pv. *gardneri* ATCC 19865^PT^ and *X*. *hortorum* B07‐007 have three types of T5SS (types 5a, 5b, and 5c) (Alvarez‐Martinez et al., 2021).

The type VI secretion system (T6SS) is mostly responsible for bacterial antagonism, thus playing an important role in competition (Bayer‐Santos et al., 2019; Russell et al., 2014). In *Xanthomonas* spp., three subclasses of T6SSs have been reported (T6SS‐I, T6SS‐II, and T6SS‐III) (Alvarez‐Martinez et al., 2021; Bayer‐Santos et al., 2019; Timilsina et al., 2020). *X*. *hortorum* pvs *hederae* WHRI 7744, *gardneri* ATCC 19865^PT^, and *cynarae* CFBP 4188^PT^ do not possess T6SS‐I and T6SS‐III clusters. A complete T6SS‐II cluster was detected in *X*. *hortorum* pv. *hederae* WHRI 7744, but not in strains CFBP 4188^PT^ and ATCC 19865^PT^ (Timilsina et al., 2020). However, two different studies reported that no T6SS was found in *X*. *hortorum* pv. *gardneri* and *X*. *hortorum* (unspecified pathovar). In one study, strain numbers were not specified (Bayer‐Santos et al., 2019), while in the other, the two strains were *X*. *hortorum* pv. *gardneri* ATCC 19865^PT^ and *X*. *hortorum* B07‐007 (Alvarez‐Martinez et al., 2021).

### 8.3 Copper resistance and homeostasis

Copper resistance is attributed to the acquisition of a copper resistance gene cluster through horizontal gene transfer (Behlau et al., 2008; Bender et al., 1990; Cooksey, 1994). Copper resistance is usually plasmid encoded (Stall et al., 1986) and can thus be acquired via conjugation by other bacteria (Basim et al., 1999). Because copper‐based solutions have been extensively used for controlling bacterial spot diseases, with recommendations going back to the 1920s (Abrahamian et al., 2020; Higgins, 1922; Obradovic et al., 2008), copper‐resistant strains pose a challenge for disease management (see *Disease control and management*).

*X. hortorum* pv. *gardneri* strains differed in their response to copper. For example, strain ATCC 19865^PT^ has *copLAB* homologs on the chromosome (*cohLAB*) and is homeostatic to copper, growing in copper concentrations up to 75 mg/L (Potnis et al., 2011). In contrast, strains JS749‐3 and ICMP 7383 have plasmidborne *copLAB* and *copMGCDF* genes (Richard, Boyer, et al., 2017; Richard, Ravigné, et al., 2017), as well as *cusAB*/*smmD* systems, involved in heavy metal efflux resistance and originally described in *Stenotrophomonas maltophilia* (Crossman et al., 2008). Strains JS749‐3 and ICMP 7383 are copper‐resistant and can grow in copper concentrations up to 470 mg/L (Richard, Ravigné, et al., 2017).

## 9 MOLECULAR HOST–PATHOGEN INTERACTIONS

The interactions of *Xanthomonas* species with their plant hosts involve the coordinated expression of various virulence factors (e.g., quorum sensing, effectors, avirulence genes) (Alvarez‐Martinez et al., 2021; Ryan et al., 2011; Timilsina et al., 2020). Quorum sensing, a chemical communication mechanism allowing bacteria to regulate group behaviours in response to stimulus, involves the production, release, and detection of auto‐inducers (Bassler, 1999; von Bodman et al., 2003; Miller & Bassler, 2001; Ng & Bassler, 2009; Whitehead et al., 2001). In *Xanthomonas*, the production and sensing of diffusible signal factors (DSF, e.g., α,β‐unsaturated fatty acids) (Wang et al., 2004) are regulated by genes within the regulation of the pathogenicity factors (*rpf*) cluster.

Knocking out *rpfF* and *rpfC* in *X*. *hortorum* pv. *pelargonii* Xhp305 altered in planta motility, decreased disease severity on pelargonium plants, and disrupted the plant colonization pattern (Barel et al., 2015). The resulting inability of *X*. *hortorum* pv. *pelargonii* to switch back and forth between biofilm and planktonic lifestyles is thus DSF‐dependent (Barel et al., 2015), and this shift is essential for pathogenicity (He & Zhang, 2008). Furthermore, in the *rpfF* and *rpfC* mutants, genes *gumM*, *pilC*, and *pilT* were down‐regulated compared to the wild type, suggesting that *gumM* expression and biofilm production, and the type 4 pilus apparatus are DSF‐dependent in *X*. *hortorum* pv. *pelargonii* (Barel et al., 2015).

Effectors are used by *Xanthomonas* species to trigger or suppress host defence mechanisms. Repertoires of effectors (effectomes) have been suggested to play a role determining host specificity (Hajri et al., 2009). Within *X*. *hortorum*, effector‐related work is mainly focused on pv. *gardneri*, but there are also reports on pv. *carotae* strains (more information below). The T3SS of *X*. *hortorum* pv. *gardneri* ATCC 19865^PT^ was associated with *hrpW* (Potnis et al., 2011), a gene predicted to encode a pectate lyase (White et al., 2009), involved in plant tissue maceration and rotting (Collmer & Keen, 1986). The function of effector gene *xopZ2*, located downstream of *hrpW*, was suggested by *avrBs2* reporter gene fusion (Potnis et al., 2011). Other T3SS effectors (T3Es) in *X*. *hortorum* pv. *gardneri* strains were also reported: XopAM, XopAO (homolog of AvrRpm1 from *P. syringae*), XopAQ (homolog of Rip6/Rip11 from *R. solanacearum*), and XopAS (homolog of HopAS1 from *P*. *syringae*). Effectors XopAM and XopAO were demonstrated to be dependent on the T3SS using the AvrBs2 reporter system (Potnis et al., 2011). In addition, four novel T3Es were reported in multiple field strains of *X*. *hortorum* pv. *gardneri*: a second XopE2 paralog, in addition to XopJ and two predicted effectors, named T3EP and PTP, with homologs in *R*. *solanacearum* and *X*. *campestris* pv. *campestris*, respectively (Schwartz et al., 2015).

Effector AvrHah1, a transcription activator‐like (TAL) effector of the AvrBs3/PthA family (Schornack et al., 2008), was the first characterized effector of *X*. *hortorum* pv. *gardneri*. AvrHah1 was able to trigger a *Bs3*‐dependent hypersensitive response (HR) on pepper plants (Schornack et al., 2008). Gain‐of‐function experiments with a *X*. *euvesicatoria* pv. *euvesicatoria* strain revealed that *avrHah1* is responsible for enhanced water‐soaking in pepper leaves, a phenotype typical for the compatible interaction of *X*. *hortorum* pv. *gardneri*, the donor pathogen (Schornack et al., 2008). The virulence function of AvrHah1, triggering enhanced water‐soaking in its known hosts tomato, pepper, and *Nicotiana benthamiana*, was attributed to the movement of water into the infected apoplast (Schwartz et al., 2017). Gene *avrBs7* was also identified in *X*. *hortorum* pv. *gardneri* as another avirulence gene as its product triggered an HR in *Capsicum baccatum* var. *pendulum* (Potnis et al., 2012). When the corresponding single dominant resistance gene *Bs7* was introgressed into *C*. *annuum* ‘Early Calwonder’ (ECW), the resulting near‐isogenic line ECW‐70R was resistant to strains harbouring *avrBs7*.

Twenty‐one candidate T3E genes were identified in *X*. *hortorum* pv. *carotae* M081, and the products of two of them, AvrBs2 and XopQ, were found to elicit effector‐triggered immunity (Kimbrel et al., 2011). Using *Agrobacterium*‐mediated transient expression of *avrBs2* from *X*. *hortorum* pv. *carotae* in transgenic *N. benthamiana* triggered an HR in a *Bs2*‐dependent manner. In contrast, no phenotypes were visible in wild‐type *N*. *benthamiana* lacking *Bs2* on delivery of the same DNA construct. Transient expression of *xopQ* also resulted in strong and rapid HRs in most of the infiltrated leaves of wild‐type *Nicotiana* *tabacum*, perhaps mediated by another resistance gene. These observations indicated a possibility for resistance gene‐mediated control of *X*. *hortorum* pv. *carotae* (Kimbrel et al., 2011). A core *Xanthomonas* effectome of nine effectors, including AvrBs2, XopQ, and XopZ previously described, was reported in the tested strains of a study including *X*. *hortorum* B07‐007 and *X*. *hortorum* pv. *gardneri* ATCC 19865^PT^.

## 10 DISEASE CONTROL AND MANAGEMENT

An integrated control programme that focuses on excluding, reducing, or eradicating the pathogen, in combination with various methods like biological control and host resistance breeding, is the most suitable to manage bacterial spot pathogens like *X*. *hortorum* (Agrios, 2005; Marin et al., 2019). Preventing *X*. *hortorum* infections by excluding the pathogen from its hosts is crucial, especially because global trade of plants, seeds, and other propagating material plays an important role in the dissemination of this species (see *Epidemiology*).

Because *X*. *hortorum* pv. *gardneri* is locally present in the territory of the European and Mediterranean Plant Protection Organization (EPPO), the pathogen is on the EPPO A2 list and is recommended for regulation as a quarantine pest (EPPO, 2021). Since 2020, the EU (European Union) Plant Health Law regulates this pathovar as a nonquarantine pest (RNQP; Picard et al., 2018) on seeds, propagating, and planting material of tomato and peppers as well as propagating material of ornamental peppers (EU Commission, 2019). Other Regional Plant Protection Organizations (RPPOs) can implement regional phytosanitary regulations. For example, *X*. *hortorum* pv. *carotae* is considered an A1 plant pest by the Caribbean Plant Protection Commission (CPPC) and is under strict quarantine control there. Certification programmes propose requirements for production of disease‐free plants. For example, certification scheme EPPO PM4/3 outlines various testing methods for *X*. *hortorum* pv. *pelargonii* on different propagation materials (nuclear, basic stock, and certified cuttings). Because cool temperatures during propagation suppress symptom expression, methods to detect low pathogen numbers in asymptomatic tissue are thus crucial (see *Identification and detection*).

Physical or chemical treatment of the planting material can decrease pathogen inoculum (Janse & Wenneker, 2002). Hot‐water seed treatment reduced *X*. *hortorum* pvs *carotae* and *gardneri* infections. However, hot‐water seed treatment can sometime be unsuitable. For example, a treatment at 50°C for 2 h of lettuce seeds against *X*. *hortorum* pv. *vitians* significantly reduced seed germination (Carisse et al., 2000). Some chemical seed treatments against this pathovar, such as soaking in 1% sodium hypochlorite for 5 min (Carisse et al., 2000), in 3% hydrogen peroxide for 5 min, or in suspensions of copper hydroxide plus mancozeb (Pernezny et al., 2002), were more effective in reducing seed contamination than others (copper hydroxide alone, benzoyl peroxide, or calcium peroxide). The seed treatments described above are limited to university or extension research and, to the best of our knowledge, are not found in official documents by the National Seed Health System (NSHS) or the International Seed Federation (ISF‐ISHI). The use of seed treatments remains at the discretion of seed production companies, which must indicate what treatment was used on each seed lot.

Management of epiphytic *X*. *hortorum* (see *Epidemiology*) is challenging. Suppression methods of epiphytic *X*. *hortorum* pv. *carotae* on carrot foliage include sanitation and the use of drip irrigation to avoid wetting the phyllosphere during seed maturation (du Toit et al., 2005). Crop rotations or fallow periods could be used to eliminate contamination in plant debris by overwintering pathovars (Barak et al., 2001). In addition, good weed control and removing diseased plants can reduce inoculum amount (Barak et al., 2001; Toussaint et al., 2012), keeping in mind that some *X*. *hortorum* pathovars can survive epiphytically on weeds, and even infect them (see *Epidemiology*). Another good practice for decreasing the risk of disseminating *X*. *hortorum* pv. *pelargonii* involves not growing perennial *Geranium* spp. near greenhouse facilities producing *Pelargonium* spp. (Nameth et al., 1999).

Foliar applications of copper‐based bactericides have been used for *X*. *hortorum* pvs *carotae* and *vitians* with variable efficacy depending on various factors (e.g., application time, disease development stage, and climate) (Bull & Koike, 2005; du Toit et al., 2005). Copper‐based applications are unsustainable as they have adverse environmental effects, and because copper‐induced resistant strains are problematic for sustainable, long‐term control (Fishel, 2005; Husak, 2015; Willis & Bishop, 2016). For example, copper‐induced resistance was reported in strains of *X*. *hortorum* pv. *gardneri* (Abbasi et al., 2015; Khanal et al., 2020). The use of nanoparticles was studied to manage copper‐resistant and/or copper‐tolerant strains. For example, silver (Ag) nanoparticles merged in a double‐stranded DNA–graphene oxide matrix (Ag‐dsDNA‐GO) exhibited antibacterial activity against copper‐tolerant *X*. *hortorum* pv. *gardneri* strains (Strayer, Oscoy, et al., 2016).

Biological control solutions against some *X*. *hortorum* pathovars have been proposed. For example, alternative nontoxic compounds that induce a systemic acquired resistance (SAR) in the host plant can provide a more environmentally sustainable approach to disease management than pesticides. The compound acibenzolar‐*S*‐methyl (ASM), a benzothiadiazole, released in Europe as Bion (Syngenta Ltd) and in the United States as Actigard 50WG (Syngenta Crop Protection Inc.), has shown positive results for controlling bacterial spot caused by *X*. *hortorum* pvs *gardneri*, *pelargonii*, and *carotae* (regarding the latter, the application was only successful on seeds) (Blainski et al., 2018; Pontes et al., 2016; Simmons et al., 2010; Yellareddygari et al., 2013). However, a limitation of ASM is its adverse effect on tomato growth and yield, which may be attributed to the energy cost associated with resistance induction (Romero et al., 2001).

Treating geranium leaves with methyl jasmonate inhibited multiplication of *X*. *hortorum* pv. *pelargonii* (Zhang, Grefer, et al., 2009) and spraying leaves with EPSs from *Lactiplantibacillus plantarum* triggered a local induced resistance in tomato leaves against *X*. *hortorum* pv. *gardneri* (Blainski et al., 2018). When tested on agar plates, various essential oils inhibited *X*. *hortorum* pathovars. *Origanum compactum* (oregano) and *Thymus vulgaris* (thyme) inhibited *X*. *hortorum* pv. *pelargonii* (Kokoskova & Pavela, 2006), and three oregano species (*O. acutidens*, *O. rotundifolium*, and *O. vulgare*) seemed to inhibit the growth of *X*. *hortorum* pvs *pelargonii* and *vitians* (Dadasoglu et al., 2011). Geraniin, a tannin extracted from sugar maple, resulted in high mortality of *X*. *hortorum* pv. *vitians* bacterial cells when tested by plate counting (Delisle‐Houde et al., 2021).

Two *P. syringae* pv. *syringae* isolates, 422 and 17‐049, decreased the colonization of *X*. *hortorum* pv. *carotae* on carrot leaves (Belvoir et al., 2019). Less virulent quorum‐sensing mutants that elicit plant SAR might have a potential in management of *X*. *hortorum* pv. *pelargonii* (Barel et al., 2015). Bacteriophages applied as foliar sprays provided significant control of the disease caused by *X*. *hortorum* pv. *gardneri* in field trials (Balogh et al., 2003; Flaherty et al., 2000). However, bacteriophage effectiveness strongly depended on UV radiation and other environmental factors that affect their persistence in the phyllosphere (Iriarte et al., 2007). In greenhouse trials on potted geraniums, daily foliar sprays of bacteriophages significantly reduced disease caused by *X*. *hortorum* pv. *pelargonii* (Flaherty et al., 2001).

## 11 HOST RESISTANCE

Resistance breeding research against *X*. *hortorum* has been focused on tomato, pepper, lettuce, carrot, and pelargonium, and multiple plant cultivars showed moderate to high resistance against the various pathovars. Wild tomatoes have a broad‐spectrum resistance against multiple *X*. *hortorum* pv. *gardneri* strains (Liabeuf et al., 2015). Screening of *S. lycopersicum* and *Solanum pimpinellifolium* germplasm using HR identified partially resistant *S*. *lycopersicum* lines, as well as three *S. pimpinellifolium* accessions (LA2533, LA1936, and PI 128,216) resistant against the pathovar. This resistance may be controlled by one to four loci with moderate heritability. The *S*. *pimpinellifolium* lines were also resistant under field conditions (Liabeuf et al., 2015). Another wild tomato genotype, *S*. *lycopersicum* var. *cerasiforme* line PI 114490, possessed broad‐spectrum resistance to multiple xanthomonad pathogens, including *X*. *hortorum* pv. *gardneri*. Resistance in PI 114490 is quantitatively inherited, and QTL‐3 locus and allele at QTL‐11 are major contributors of resistance against *X*. *hortorum* pv. *gardneri* (Bernal et al., 2020).

A mutation in *DMR6* (downy mildew resistance 6) in *Arabidopsis*, conferring broad‐spectrum resistance to various *Xanthomonas* and *Pseudomonas* phythopathogens, was tested in tomato (Thomazella et al., 2016). The stable transgenic tomato plants were resistant against *X*. *hortorum* pv. *gardneri* and were not compromised in their growth and development.

In pepper, two dominant resistance genes, *Bs3* and *Bs7*, are known to confer resistance against *X*. *hortorum* pv. *gardneri* strains carrying avirulence genes *avrHah1* and *avrBs7*, respectively (Potnis et al., 2012; Schornack et al., 2008). However, the plasmidborne nature of both avirulence genes suggests vulnerability to resistance breakdown, so they have not been further considered in breeding programmes. Screening of core pepper germplasm collection against *X*. *hortorum* pv. *gardneri* revealed that more than 40 PI lines of *C*. *baccatum* in greenhouse conditions and multiple PI lines of *C*. *annuum* showed promising resistance levels (Potnis et al., 2012). A total of 20 significant single nucleotide polymorphisms (SNPs), co‐located within 150 kb of 92 unique genes, were recently identified against the pathovar (Potnis et al., 2019).

Regarding *X*. *hortorum* pv. *vitians*, different lettuce genotypes (*L*. *sativa*) show differential responses to the pathogen. For example, romaine and butterhead lettuce cultivars are among the highly susceptible ones (Pernezny et al., 1995). Moderately resistant cultivars include both green‐leaf (e.g., Waldmann's Green and Grand Rapids) and red‐leaf (e.g., Red Line) cultivars (Carisse et al., 2000), although other studies have noted their susceptibility (Bull et al., 2007). Such discrepancy could be due to differences in the experimental setups of the studies, such as using different strains for the pathogenicity tests. Other moderately resistant cultivars include Little Gem and Reine des Glaces (Batavia crisphead) (Bull et al., 2007). These two latter cultivars were deemed to be promising in breeding resistant cultivars against *X*. *hortorum* pv. *vitians* (Hayes, Trent, Mou, et al., 2014; Hayes, Trent, Truco, et al., 2014). However, undesirable traits (e.g., small size and low yield) associated with cv. Little Gem are of concern. Furthermore, this cultivar has also shown variable resistance in separate studies, making it an unattractive candidate (Bull et al., 2007; Lu & Raid, 2013). This difference could be due either to virulence dissimilarities at the strain level or to host susceptibility variation as a result of different environmental conditions at the cultivar evaluation locations (Lu & Raid, 2013). In addition, resistance of cv. Reine des Glaces was also highly dependent on environmental conditions (Bull et al., 2007).

Genetic maps of various wild lettuce species like *L*. *serriola*, *L. saligna*, and *L. virosa*, have revealed multiple genes conferring broad resistance (McHale et al., 2009; Truco et al., 2013). However, *L*. *saligna* and *L*. *virosa* have compatibility issues, making hybridization difficult. The broad resistance in wild lettuce species have yet to be tested against *X*. *hortorum* pv. *vitians*.

The high genetic variability of the pathogen population is a challenge for breeding cultivars with durable resistance. Resistance against MLSA‐based groups B, D, and E of *X*. *hortorum* pv. *vitians* was identified to be controlled by a single dominant locus, *Xanthomonas resistance 1* (*Xar1*), in the Batavia heirloom cv. La Brillante (Bull et al., 2016; Hayes, Trent, Mou, et al., 2014; Hayes, Trent, Truco, et al., 2014). Two other cultivars, Little Gem and Pavane, carry *Xar1* alleles and are resistant to Californian isolates of *X*. *hortorum* pv. *vitians*. Another locus identified as *X*. *campestris vitians resistance* (*Xcvr*) was found in the same linkage group (LG2) during the mapping of a PI 358001‐1 × Tall Guzmaine population. The durability of *Xar1* and *Xcvr* resistances in cv. La Brillante and PI 358001‐1 raised concerns because of the high variability in the pathogen population (Hayes, Trent, Mou, et al., 2014; Hayes, Trent, Truco, et al., 2014). Major and minor quantitative trait loci (QTLs) controlling this resistance were identified and co‐located in the same region of LG2 as previously identified with *Xar1* and *Xcvr* (Sandoya et al., 2019).

A germplasm screening of carrot species (e.g., PI lines, public inbred lines, commercial cultivars, and wild varieties) indicated four PI lines (PI 263601, PI 418967, PI 432905, and PI 432906) and two of the wild relatives, Ames 7674 and SS10 OR, were the most resistant against *X*. *hortorum* pv. *carotae* (Christianson et al., 2015). The resistant PI lines are promising for use in commercial breeding programmes (Christianson et al., 2015).

In the genus *Pelargonium*, only a small number of pelargonium and geranium species have been screened for resistance against *X*. *hortorum* pv. *pelargonii* based on symptom expression alone after pathogen inoculation (Griesbach & Olbricht, 2002; Zhang, Sairam, et al., 2009). Five resistant pelargonium species were identified (Griesbach & Olbricht, 2002; Zhang, Sairam, et al., 2009), but most commercially important cultivars of *Pelargonium zonale* hybrids were highly susceptible (Griesbach & Olbricht, 2002; Zhang, Sairam, et al., 2009).

## 12 RESEARCH PERSPECTIVES

Several advances improving our understanding of the *X*. *hortorum* species have recently been published. However, some knowledge gaps remain, mainly related to extent of host range, detection, and control methods, including host resistance. Given the broad and diverse host range described for this species, it is likely that unreported hosts remain to be identified in various ecosystems. Further investigation of the natural and experimental host ranges of *X*. *hortorum* could provide insight into its evolutionary history and determine if plant domestication influenced host specialization of the pathovars of *X*. *hortorum*.

The recent increased availability of genomic data for *X*. *hortorum* will help in the identification of novel isolates from new natural hosts through establishing quick, field‐deployable detection methods. Such tools will also be very beneficial for phytosanitary control, especially as prevention strategies are preferred to formulation applications and are less costly than containment and eradication measures.

There is a significant need to conduct a comprehensive comparative genomics analysis of this species, especially in view of the recent taxonomical changes. Because plasmids offer a potentially large source of variation in the species, determining the plasmid content of strains and their contribution to pathogenicity is highly relevant. The recent application of a TnSeq analysis in *X*. *hortorum* pv. *vitians* paves the way to functional genomics analysis of other *X*. *hortorum* members. Aside from providing insights into essential bacterial genes in different in vitro and in planta conditions, TnSeq would also considerably improve our understanding of *X*. *hortorum* biology.

Most important commercial varieties are still highly susceptible to diseases caused by *X*. *hortorum*. The inefficiency of application‐based control strategies further consolidates host resistance as a promising area for devising practical and durable disease control solutions against *X*. *hortorum*. Because nonhost resistance is more durable than host resistance, screening more nonhost species for their disease response to *X*. *hortorum* could uncover broad, nonhost resistance genes against the pathovars. Furthermore, exploring recent advancements in the field of host resistance against bacteria, such as CRISPR/Cas9‐mediated gene mutations, also sound promising for breeding *X*. *hortorum*‐resistant plant cultivars. However, as highlighted throughout this pathogen profile, the high genetic variability of these phytopathogens affecting several plant families represents a real challenge for long‐term resistance.

## CONFLICT OF INTEREST

The authors declare that they have no competing interests.

## Data Availability

Data sharing is not applicable to this article as no new data were created or analysed in this study.
